# Seasonal Change in Microbial Diversity and Its Relationship with Soil Chemical Properties in an Orchard

**DOI:** 10.1371/journal.pone.0215556

**Published:** 2019-12-31

**Authors:** Xuhui Luo, Ming Kuang Wang, Guiping Hu, Boqi Weng

**Affiliations:** 1 Agricultural Ecology Institute, Fujian Academy of Agricultural Sciences, Fuzhou, Fujian Province, China; 2 Department of Agricultural Chemistry, National Taiwan University, Taipei, Taiwan; 3 Jiangxi Sericulture and Tea of Research Institute, Nanchang, Jiangxi Province, China; 4 Fuzhou Scientific Observing and Experimental Station of Agro-Environment, Ministry of Agriculture and Rural Affairs of People’s Republic China, Fuzhou, Fujian Province, China; 5 Fujian Key Laboratory on Ecological Processes of Hilly Agriculture in Red Soil Region, Agency of Fujian Science and Technology, Fuzhou, Fujian Province, China; Guangdong Technion Israel Institute of Technology, CHINA

## Abstract

This study aimed to determine the microbial diversity at different soil depths (0–5 and 5–20 cm) in a subtropical orchard during different seasons (i.e., spring, summer and autumn) to advance knowledge of the roles of microbes in orchard ecosystem balance. In tracking experiments conducted in an orchard (established in 1996), the phospholipid fatty acid (PLFA) biomarker method was employed to determine the soil microbial system. The total PLFA concentration did not vary significantly between soil depths but changed between seasons. It peaked in the summer at 258.97 ± 23.48 μg g soil^-1^ from 0–5 cm and at 270.99 ± 58.94 μg g soil^-1^ from 5–20 cm. A total of 33 microbial fatty acid biomarkers were observed and identified in the sampled soil. The quantities of PLFAs for 29 microbial groups varied significantly between seasons, except for 15:0 iso 3OH, 15:1 iso G, 16:0 2OH, and 17:0 iso 3OH. The bacterial PLFAs and fungal and actinomycetic PLFAs in the orchard soil collected in summer were significantly more abundant than those collected in the spring or autumn (*P* < 0.01). The number of soil microorganism species (richness) and the Simpson and Shannon-Wiener indexes were all highest in summer. The total PLFAs, bacterial PLFAs, fungal PLFAs, actinomycetic PLFAs, richness, and Simpson and Shannon-Wiener indexes were all significantly negatively correlated with soil pH, total organic carbon (TOC), total nitrogen (TN) and the cation exchange capacity (CEC) (*P* < 0.05).

## Introduction

The soil is alive in that hundreds of millions or even billions of microbes reproduce, grow and die in one gram of soil. This complex community acts as a changeable nutrient pool, which affects the degree to which additional nutrients are utilized by trees and grasses and the degree to which they are emitted into the environment. Recent research has revealed that changes in this pool are driven not only by artificial management but also by annual seasonal variation. Using the method of phospholipid fatty acid (PLFA) analysis to define the relationship between the live microbial community and soil chemical properties in the context of seasonality could help us learn more about soil microorganism functions.

Usually, this relationship is more difficult to make clear in agricultural ecosystems than in forest and grassland ecosystems because the soil-microbe interactions in the former related to management as well as seasonal moisture and temperature. However, it is urgently necessary to clarify agricultural soil-microbe interactions in order to maintain their delicate balance and reduce ecological problems, such as biodiversity decreases [1], soil erosion [2], and pollution [3,4]. Additionally, such clarification is very useful because soil microbes play a unique and indispensable role in the agricultural ecosystem balance [5].

Orchard ecosystems are important for fruit production and carbon sequestration, and the soil microbial community changes with soil quality because of long-term management. This relationship is reflected by effects on microbial diversity in terms of chemical property changes [6–11], and the spatial and temporal distributions of the microbial community help explain the interaction [12–18]. However, little clear information is available regarding the temporal distribution of the microbial community in subtropical orchards.

Thus, we hypothesized that changes in height, seasonal temperature, moisture variation and vertical soil chemical properties would lead to obvious differences in the spatial and temporal distributions of the microbial community in subtropical orchards, especially in Fujian Province, which is one of the major areas used for fruit production. The seasonal (spring, summer and autumn) and vertical changes (0–5 and 5-20-cm soil depths) in microbial diversity and the links between soil microbial diversity and soil chemical properties in subtropical orchards remain obscure. PLFA analysis was employed to monitor microorganism quantity and diversity in orchard soils in different seasons in the hilly red soil of the subtropical zone in southern China. The observations of this study regarding the ecological parameters of orchard soil microorganisms can provide a scientific basis for further studies and management strategies to improve fruit production.

## Materials and methods

### 2.1. Experimental area

**Fig 1:**
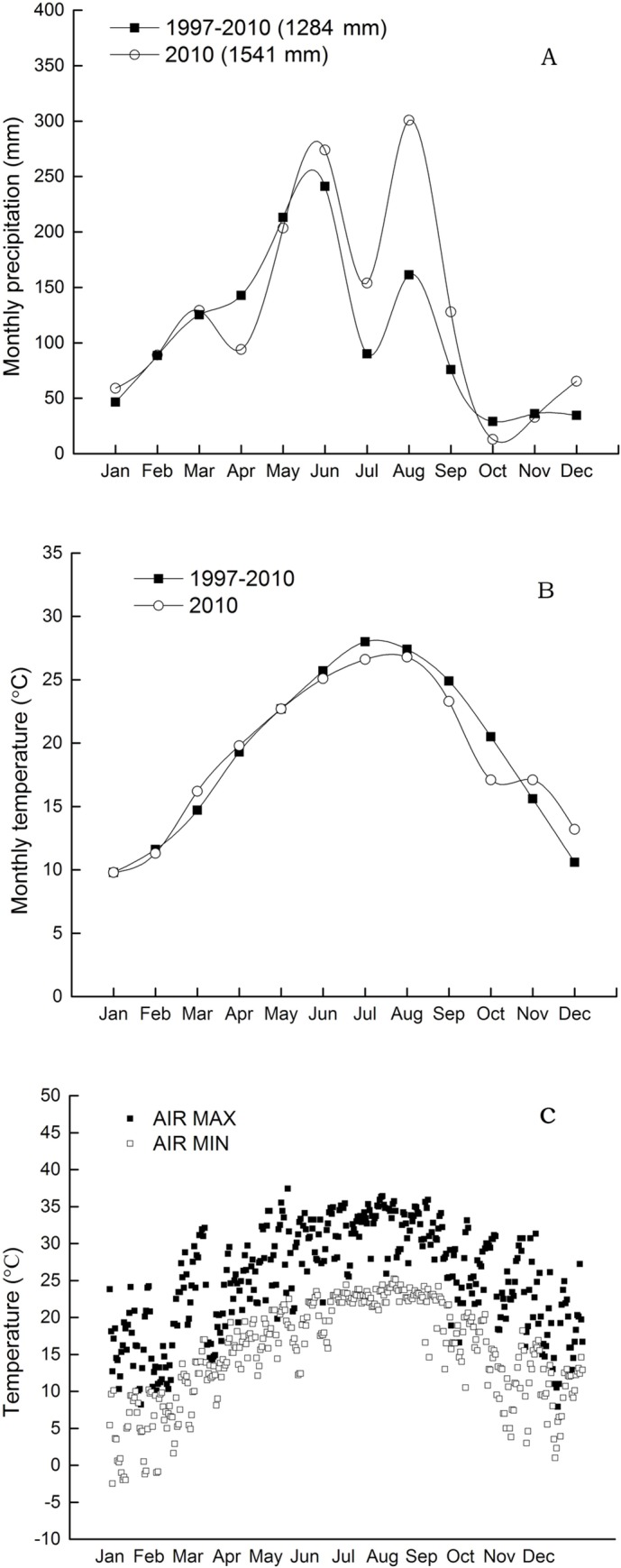
Monthly dynamics of precipitation (A), average air temperature (B), and extreme air temperature (C) at the trial location from 1997 to 2010. Annual precipitation is shown in parentheses (A).

The experimental area was located at the Yuchi Village Experimental Station, Xicheng Township, Youxi County, Fujian Province, southeastern China (26° 25' N, 117° 57' E). The area has a subtropical humid monsoon climate with an annual sunshine time of 1,781.7 h (accounting for 40% of the available annual sunshine hours); an annual precipitation of 1,284 mm (Fig 1); an annual average temperature of 19.2°C; an average temperature in July of 26.6–28.9°C and in January of 8.0–12.0°C; and a frost-free period of more than 312 d. The experimental station was established in 1996 as a peach orchard water and soil conservation monitoring system with an altitude of 150 m and a slope of 15° facing south-southeast. The soil is a Quaternary-aged red soil with a clay texture [19]. The experimental field was originally a secondary shrub-barren hill with the plant species *Dicranopteris dichotoma* Bernh, *Miscanthus floridulus* War ex Schum, and *Miscanthus sinensis* Anderss. The surrounding vegetation was mainly coniferous and coniferous-deciduous-bamboo mixed stands with *Pinus massoniana* Lamb and *Cunninghamia lanceolata* Hook and a grove of *Phyllostachys heterocycla* cv. pubescens. The station was initially established for fixed soil erosion monitoring.

### 2.2. Soil samplings

Spring, summer and autumn were the seasons examined. On April 27, August 22, and November 4, 2010, which were chosen because they occurred 3 d after rain (to keep soil moisture at the same level), soils were sampled at depths of 0–5 and 5–20 cm from five random locations in the unfertilized zones in the area surrounding mean sample trees. The soil temperature during sampling is listed in Table 1, with an average temperature in spring of 21.3–22.0°C, in summer of 27.0–28.0°C and in autumn of 18.0–20.2°C. The air moisture measured at the same time was 82% for spring, 86% for summer, and 82.6% for autumn. After collection, the soils from the same soil layer were mixed well in the field, sealed in bags, and immediately brought to the laboratory for measurement or stored in a -80°C freezer for soil microbial analysis. Other mixed soil samples were immediately carried to the laboratory for water content testing and air-dried for basic chemical property analysis.

**Table 1:** Soil temperature and air moisture at different sampling stages in the orchard.

Item	Spring	Summer	Autumn
Soil temperature (°C)			
0 cm	21.3	27.5	18.0
5 cm	22.0	27.7	19.0
10 cm	21.5	28.0	19.5
15 cm	21.7	27.3	20.2
20 cm	21.4	27.0	19.7
Air relative moisture (%)	82.0	86.0	82.6

Soil temperature and air moisture are the averages of two weeks of data before sampling on April 27 for spring, on August 22 for summer and on November 4 for autumn.

### 2.3. Chemical analysis

Soil pH was measured in deionized water (1:5, soil:water). The total organic carbon (TOC) and total nitrogen (TN) were determined by potassium dichromate [20] and Kjeldahl digestion-distillation [21]. Exchangeable cations (K^+^, Na^+^, Mg^2+^ and Ca^2+^) were extracted by CH_3_COONH_4_ solution (pH = 7.0) [22] and analyzed using atomic absorption spectrophotometry (AA-6800, Shimadzu Corp., Kyoto, Japan). Soil samples were as described above and conducted in triplicate.

### 2.4. Extraction and determination of microbial PLFAs

Soil PLFA extraction involved four steps: 1) Five grams of soil was placed in a centrifuge tube. Then, 15 mL of a 0.2 M KOH (Sinopharm Chemical Reagent Co., Ltd., Shanghai, China) and methanol (Fisher Scientific Worldwide (Shanghai) Co., Ltd., Shanghai, China) solution was added before tightening the cap. The centrifuge tube was shaken for 5 min at 100 rpm. After shaking, the tube was incubated in a CU600 thermostat water bath for 5 min at 37°C. This procedure was repeated five times to help release the fatty acids from the soil sample. 2) Then, the tube was opened, and 3 mL of 1.0 M acetic acid (Sinopharm Chemical Reagent Co., Ltd) solution was added to decrease the pH of the reaction. 3) After adding 10 mL of n-hexane (Merck Co., Darmstadt, German) and mixing well, the tubes were centrifuged in an N00077 centrifuge for 15 min using the following settings: rotor: #12,150; speed: 2,000 rpm; time: 15 min; and temperature: 4°C. After centrifugation, the n-hexane supernatant was transferred to a clean flask and air-dried under a fan. 4) The air-dried sample was resuspended in 0.5 mL of a mixture of n-hexane:methyl-tert-butyl ether (Tedia Co., Inc., Fairfield, OH) (1:1, v/v) for 3–5 min and transferred to gas chromatography (GC) vials for PLFA determination.

Microbial PLFAs were determined using the Sherlock Microbial Identification System (MIS) 4.5 (MIDI Inc., Newark, DE), which included a 6890N GC system (Agilent Technologies Inc., Palo Alto, CA), automatic injection devices, a quartz capillary column, and a flame ionization detector. The standard PLFA-methyl ester (MIDI, Inc.) mixture and extracted samples were analyzed under the following chromatographic conditions: temperature increment: controlled by the second-order program, with an initial temperature of 170°C that increased to 260°C at 5°C min^-1^ and then to 310°C at 40°C min^-1^ and was maintained at 310°C for 90 sec; vaporization chamber temperature: 250°C; detector temperature: 300°C; carrier gas: H_2_ (2 mL min^-1^); blowing gas: N_2_ (30 mL min^-1^); precolumn pressure: 68.95 kPa; injection volume: 1 μL; injection split ratio: 100:1; and ionization mode: electron ionization (EI).

### 2.5. Calculation of microbial diversity

Known concentrations of 19:0 (nonadecanoic methyl ester) were added as internal standards and used to convert the retention-time peak areas to nanomoles per gram (nmol g^-1^) of soil (absolute abundance) and mole percent (mol %) (proportional abundance) of liquids. The absolute and proportional abundances of specific microbial groups were calculated by summation of diagnostic lipid markers. The sum of 16:1 ω9c (PLFA configuration type), 18:1 ω9c and 18:3 ω6c (6, 9, 12) was used to indicate fungi. The sum of 17:0 10 methyl, 18:0 10 methyl, 18:1 ω9c and 18:3 ω6c (6, 9, 12) was used to indicate actinomycetes. The sum of 12:0, 14:0, 14:0 anteiso, 14:0 iso, 15:0 2OH, 15:0 3OH, 15:0 anteiso, 15:0 iso, 15:0 iso 3OH, 15:1 iso G, 16:0, 16:0 10 methyl, 16:0 2OH, 16:0 anteiso, 16:0 iso, 16:1 ω5c, 17:0 anteiso, 17:0 cyclo, 17:0 iso, 17:0 iso 3OH, 17:1 ω8c, 18:0, 18:0 iso, 18:1 ω7c, 18:1 ω7c 11 methyl, 18:3 ω6c (6,9,12), 19:0 cyclo ω8c, 19:0 iso, and 20:0 was used to indicate bacteria [23].

The number of species (richness), Simpson diversity, Shannon-Wiener diversity and Alatalo evenness were used to calculate the ecological parameters of the microbial fatty acid biomarkers. The equations were as follows:

$$\mathrm{The}\;\mathrm{Simpson}\;\mathrm{diversity}\;\mathrm{index}\;\mathrm{was}\;\mathrm{calculated}\;\mathrm{according}\;\mathrm{to}\;D=1-\sum Pi^{2},$$ (1)

TheShannon‐WienerdiversityindexcalculatedaccordingtoH=−∑Pi•ln(Pi), (2)

TheAlataloevennessindexwascalculatedaccordingtoJ=[1/∑(Pi2)−1][exp(H)−1], (3)

where Pi=NiN; Ni is the content of the i^th^ kind of PLFA; and N is the total PLFA content.

### 2.6. Statistical analysis

A one-way analysis of variance (ANOVA) followed by a least significant difference (LSD) multiple comparison test was used to detect significant differences among the means of soil properties, microbial community indicators (bacterial PLFAs, fungal PLFAs, actinomycetic PLFAs, and B/F ratio of PLFAs), and microbial diversity indicators (richness, Simpson index, Shannon-Wiener index and Alatalo index) in different seasons. A two-way analysis was used to detect significant differences in the total PLFA and individual PLFA variance between seasons and soil depths. A Spearman coefficient analysis was used to measure the correlation between soil properties and microbial indicators. A principal component analysis was used to measure the microbial community change between different seasons and soil depths. These analyses were all performed in SPSS software version 17.0 (Chicago, IL).

## Results

### 3.1. Soil chemical properties in different seasons in the orchard trials

The soils were classified as clay, thermal and Typic Hapludult [24]. The principal chemical properties of the soil samples collected in the spring, summer and autumn at depths of 0–5 and 5–20 cm are presented in Table 2. The soil pH (0–5 cm: 4.46; 5–20 cm: 4.38), TOC (0–5 cm: 12.32 g kg^-1^; 5–20 cm: 10.25 g kg^-1^), TN (0–5 cm: 1.14 g kg^-1^; 5–20 cm: 0.86 g kg^-1^), and cation exchange capacity (CEC) (0–5 cm: 4.95 cmol(+) kg^-1^; 5–20 cm: 5.34 cmol(+) kg^-1^) were lowest in the summer samples, with significant differences (*P <* 0.05). However, the exchangeable K^+^ (0–5 cm: 141.79 g kg^-1^; 5–20 cm: 99.22 g kg^-1^) and Na^+^ (0–5 cm: 30.67 g kg^-1^; 5–20 cm: 43.80 g kg^-1^) were significantly higher in summer than in the other seasons. Furthermore, the ratio of TOC to TN (C/N ratio) in the soil varied significantly between seasons, with the largest value in the spring (0–5 cm: 15.81; 5–20 cm: 12.85), an intermediate value in the summer (0–5 cm: 10.76; 5–20 cm: 11.88), and the lowest value in the autumn (0–5 cm: 5.90; 5–20 cm: 9.04) (*P <* 0.05). No significant differences in exchangeable Mg^2+^ or Ca^2+^ were detected between seasons (*P >* 0.05). No significant differences in pH, TOC, the C/N ratio, CEC, exchangeable Mg^2+^ or Ca^2+^ were found between soil depths.

**Table 2:** Chemical characteristics of soil sampled in different seasons at 0–5 and 5-20-cm soil depths in the orchard.

Item	Spring	Summer	Autumn
0–5 cm			
pH	4.87±0.06 a (5)	4.46±0.08 b (5)	4.95±0.04 a (5)
TOC (g kg^-1^)	20.90±1.07 a	12.32±1.53 b	20.92±1.17 a
TN (g kg^-1^)	1.46±0.24 bc	1.14±0.08 c	3.95±0.75 a
C/N ratio	15.81±3.30 a	10.76±0.93 b	5.90±1.18 c
CEC (cmol(+) kg^-1^)	9.42±0.26 a	4.95±0.29 c	7.09±0.25 b
Exchangeable K^+^ (mg kg^-1^)	124.00±15.64 a	141.79±30.11 a	99.67±13.16 b
Exchangeable Na^+^ (mg kg^-1^)	17.75±0.62 b	30.67±3.61 a	14.09±1.13 b
Exchangeable Mg^2+^ (mg kg^-1^)	30.14±3.83 a	28.41±7.56 a	24.80±2.79 a
Exchangeable Ca^2+^ (mg kg^-1^)	163.98±20.25 a	122.72±20.74 a	192.83±33.25 a
5–20 cm			
pH	4.81±0.06 a (5)	4.38±0.07 b (5)	4.83±0.08 a (5)
TOC (g kg^-1^)	19.15±0.92 a	10.25±0.89 b	18.78±0.67 a
TN (g kg^-1^)	1.61±0.14 ab	0.86±0.06 b	2.20±0.25 a
C/N ratio	12.85±0.81 ab	11.88±0.55 b	9.04±1.10 bc
CEC (cmol(+) kg^-1^)	9.29±0.56 a	5.34±0.39 c	6.87±0.18 b
Exchangeable K^+^ (mg kg^-1^)	62.85±7.54 ab	99.22±12.24 a	55.95±7.85 ab
Exchangeable Na^+^ (mg kg^-1^)	15.22±1.26 b	43.80±5.91 a	10.21±0.85 b
Exchangeable Mg^2+^ (mg kg^-1^)	22.71±6.17 a	34.47±8.80 a	20.05±4.82 a
Exchangeable Ca^2+^ (mg kg^-1^)	121.38±22.12 a	117.97±51.79 a	164.97±18.31 a

TOC = total organic carbon, TN = total nitrogen, CEC = cation exchange capacity, C/N ratio = ratio of total organic carbon to total nitrogen. Values followed by the same letter(s) in a row are not significantly different at *P* < 0.05 based on LSD post hoc tests. The sample size is shown in parentheses.

### 3.2. PLFAs of total microbes, bacteria, fungi and actinomycetes in different seasons

The total microbial PLFAs peaked in the summer at both the 0–5 and 5-20-cm soil depths (0–5 cm: 258.97 μg g soil^-1^, *P <* 0.01; 5–20 cm: 270.99 μg g soil^-1^, *P <* 0.01). The results of the two-way analysis demonstrated that the total microbial PLFAs were significantly different between seasons (*P* < 0.001). The quantities of bacterial, fungal and actinomycetic PLFAs in the orchard soil were significantly larger in the summer than in the spring and autumn at the 0-5- and 5-20-cm soil depths (*P <* 0.01). At the 0-5-cm soil depth, the peak values of bacterial PLFAs, fungal PLFAs and actinomycetic PLFAs were 216.05, 33.94 and 8.96 μg g soil^-1^, respectively. At the 5-20-cm soil depth, the values of bacterial PLFAs, fungal PLFAs and actinomycetic PLFAs totaled 230.00, 31.15 and 9.83 μg g soil^-1^, respectively (Table 3). The microbial PLFA contents (μg g soil^-1^) in different seasons sampled at 0-5- and 5-20-cm soil depths in the orchard are shown in Table 4. The results of two-way ANOVA of the effects of seasons (spring, summer, and autumn) and soil depths (0–5 cm and 5–20 cm) on microbial PLFA contents are provided in Table 5. The quantities of PLFAs for 29 microbial groups varied significantly between seasons, except for 15:0 iso 3OH, 15:1 iso G, 16:0 2OH, and 17:0 iso 3OH.

**Table 3:** Total PLFAs, bacterial PLFAs, fungal PLFAs, actinomycetal PLFAs, and the ratio of bacteria to fungi of soils sampled in different seasons at 0-5- and 5-20-cm depths in the orchard.

Item	Spring	Summer	Autumn
0–5 cm			
Total PLFAs (μg g soil^-1^)	14.07±8.23 c B (4)	258.97±23.48 a A (5)	99. 93±18.62 b B (4)
Bacterial PLFAs (μg g soil^-1^)	11.46±6.75 b B	216.05±20.44 a A	79.79±14.56 b B
Fungal PLFAs (μg g soil^-1^)	2.27±1.23 c B	33.94±3.96 a A	16.09±3.64 b B
Actinomycetal PLFAs (μg g soil^-1^)	0.34±0.26 b B	8.96±0.81 a A	4.05±1.00 b B
B/F ratio	4.79±0.29 a A	6.37±0.61 a A	5.15±0.60 a A
5–20 cm			
Total PLFAs (μg g soil^-1^)	10.63±1.58 b B (5)	270.99±58.94 a A (5)	51.28±7.17 b B (5)
Bacterial PLFAs (μg g soil^-1^)	8.66±1.29 b B	230.00±50.36 a A	44.60±6.05 b B
Fungal PLFAs (μg g soil^-1^)	1.71±0.32 c B	31.15±6.26 a A	5.37±0.83 bc B
Actinomycetal PLFAs (μg g soil^-1^)	0.24±0.05 b B	9.83±2.45 a A	1.30±0.60 b B
B/F ratio	5.33±0.59 b B	7.38±0.24 a AB	8.70±1.07 a A

Mean ± standard error. The values followed by the same lowercase and capital letter(s) in a column are not significantly different at *P* < 0.05 and *P* < 0.01 based on LSD post hoc tests, respectively. The sample size is shown in parentheses. One sample was missed for spring and autumn at the 0-5-cm depth.

**Table 4:** Microbial PLFA contents (μg g soil^-1^) in different seasons at 0-5- and 5-20-cm soil depths in the orchard.

Fatty acid configuration	0–5 cm			5–20 cm		
	Spring (4)	Summer (5)	Autumn (4)	Spring (5)	Summer (5)	Autumn (5)
12:0	0.05±0.04	2.67±0.44	0.65±0.17	0.00±0.00	1.68±1.03	0.23±0.14
14:0	0.19±0.10	4.89±0.54	1.43±0.26	0.16±0.03	5.15±1.06	0.76±0.06
14:0 anteiso	0.04±0.04	0.00±0.00	0.15±0.15	0.03±0.03	0.00±0.00	0.68±0.23
14:0 iso	0.03±0.03	1.72±0.20	0.38±0.09	0.01±0.01	1.70±0.41	0.05±0.05
15:0 2OH	0.06±0.06	0.37 ±0.18	0.00±0.00	0.01±0.01	0.46±0.19	0.00± 0.00
15:0 3OH	0.19±0.14	3.99±0.88	1.14±0.37	0.18±0.04	4.33±0.86	0.61±0.30
15:0 anteiso	0.40±0.15	9.07±1.17	3.22±0.68	0.43±0.08	9.25±1.85	2.13±0.23
15:0 iso	0.58±0.38	15.08±0.91	5.44±1.15	0.22±0.07	13.97±5.74	2.74±0.86
15:0 iso 3OH	0.02±0.02	0.00±0.00	0.00±0.00	0.07±0.04	0.00±0.00	0.00±0.00
15:1 iso G	0.04±0.03	0.00±0.00	0.15±0.11	0.94±0.84	13.96±13.96	2.29±2.25
16:0	3.85±2.34	56.42±4.23	23.36±3.44	2.14±0.45	48.29±14.45	9.61±2.13
16:0 10 methyl	0.68±0.36	16.04±1.90	5.92±1.22	0.47±0.07	17.58±3.72	3.54±0.82
16:0 2OH	0.00±0.00	0.00±0.00	0.00±0.00	0.01±0.01	0.00±0.00	0.00±0.00
16:0 anteiso	0.24±0.04	4.01±1.50	1.30±0.24	0.37±0.07	2.49±0.36	0.92±0.17
16:0 iso	0.64±0.40	17.89±2.61	6.09±1.33	0.38±0.06	18.19±3.91	3.70±0.64
16:1 ω9c	0.00±0.00	1.86±0.57	2.76±0.61	0.00±0.00	1.14±0.41	0.00±0.00
16:1 ω5c	1.13±0.88	7.33±0.77	0.00±0.00	0.52±0.17	7.86±2.04	1.22±0.19
17:0 10 methyl	0.14±0.11	3.22±0.26	1.33±0.28	0.08±0.02	3.89±0.76	0.59±0.20
17:0 anteiso	0.46±0.14	8.94±1.36	3.06±0.64	0.52 ±0.11	8.70±1.52	1.78±0.17
17:0 cyclo	0.09±0.07	4.38±0.77	1.04±0.29	0.08±0.01	4.31±0.77	0.52±0.15
17:0 iso	0.48 ±0.25	17.13±2.33	5.35±1.21	0.33±0.05	18.53±3.27	3.42±0.28
17:0 iso 3OH	0.00±0.00	0.00±0.00	0.00±0.00	0.12±0.07	0.00±0.00	0.00±0.00
17:1 ω8c	0.08±0.08	2.23±0.49	0.40±0.18	0.02±0.02	1.73±0.50	0.00±0.00
18:0	0.70±0.35	12.99±1.20	5.89±1.07	0.66±0.13	13.39±2.69	3.19±0.37
18:0 iso	0.03±0.03	2.24±0.24	0.47±0.27	0.03±0.02	2.73±0.52	0.20±0.13
18:0 10 methyl	0.20±0.15	5.75±0.58	2.72±0.76	0.16±0.04	5.79±1.72	0.70±0.43
18:1 ω9c	1.93±1.06	28.87±3.45	14.79±3.42	1.44±0.30	27.09±5.88	4.57±0.74
18:1 ω7c	0.53±0.34	7.86±0.86	3.28±0.56	0.38±0.15	8.90±2.45	1.55±0.28
18:1 ω7c 11 methyl	0.08±0.08	1.66±0.14	0.58±0.42	0.00±0.00	1.56±0.48	0.00±0.00
18:3 ω6c (6,9,12)	0.33±0.17	3.21±0.80	1.30±0.23	0.28±0.03	3.06±0.40	0.79±0.24
19:0 cyclo ω8c	0.65±0.38	15.95±0.72	6.39±1.43	0.43±0.06	22.14±5.22	4.83±0.84
19:0 iso	0.01±0.01	0.53±0.32	0.00±0.00	0.01±0.01	0.44±0.14	0.00±0.00
20:0	0.20±0.10	2.67±0.10	1.34±0.24	0.16±0.04	2.66±0.62	0.63±0.12

The sample size is shown in parentheses. Mean±standard error.

**Table 5:** Two-way analysis of variance of the effects of season (spring, summer, and autumn) and soil depth (0–5 cm and 5–20 cm) on microbial PLFA contents.

Fatty acid configuration	Microbial group	Season	Soil depth	*r*^*2*^
12:0	Gram-negative bacteria	**^a^	n.s.	0.522
14:0	Gram-negative bacteria	**	n.s.	0.815
14:0 anteiso	Gram-positive bacteria	**	n.s.	0.554
14:0 iso	Gram-positive bacteria	***	n.s.	0.795
15:0 2OH	Gram-negative bacteria	**	n.s.	0.425
15:0 3OH	Gram-negative bacteria	***	n.s.	0.723
15:0 anteiso	Gram-positive bacteria	***	n.s.	0.796
15:0 iso	Gram-positive bacteria	***	n.s.	0.599
15:0 iso 3OH	Gram-negative bacteria	n.s.	n.s.	0.297
15:1 iso G	Bacteria	n.s.	n.s.	0.155
16:0	Bacteria	***	n.s.	0.733
16:0 10 methyl	Sulfate-reducing bacteria	***	n.s.	0.785
16:0 2OH	*Ralstonia* spp.	n.s.	n.s.	0.170
16:0 anteiso	Gram-positive bacteria	**	n.s.	0.508
16:0 iso	Gram-positive bacteria	***	n.s.	0.774
16:1 ω9c	Fungi	***	n.s.	0.704
16:1 ω5c	Methane-oxidizing bacteria	***	n.s.	0.613
17:0 10 methyl	Actinomycetes	***	n.s.	0.814
17:0 anteiso	Gram-positive bacteria	***	n.s.	0.806
17:0 cyclo	Gram-negative bacteria	***	n.s.	0.801
17:0 iso	Gram-positive bacteria	***	n.s.	0.806
17:0 iso 3OH	Gram-negative bacteria	n.s.	n.s.	0.352
17:1 ω8c	Gram-negative bacteria	***	n.s.	0.693
18:0	*Hydrogenobacter*	***	n.s.	0.808
18:0 iso	Gram-positive bacteria	***	n.s.	0.821
18:0 10 methyl	Actinomycetes	***	n.s.	0.691
18:1 ω9c	Fungi	***	n.s.	0.775
18:1 ω7c	*Pseudomonas* spp.	***	n.s.	0.705
18:1 ω7c 11 methyl	*Cellulomonas* spp.	***	n.s.	0.670
18:3 ω6c (6,9,12)	Fungi	***	n.s.	0.700
19:0 cyclo ω8c	*Burkholderia*	***	n.s.	0.758
19:0 iso	Bacteria in general	**	n.s.	0.375
20:0	Bacteria in general	***	n.s.	0.782

*, ** and *** indicate significant differences in a row at *P <* 0.05, 0.01, and 0.001, respectively. Nonsignificant results are labeled n.s. (*P* > 0.05).

### 3.3. Change in microbial diversity between seasons in the orchard trial

**Fig 2:**
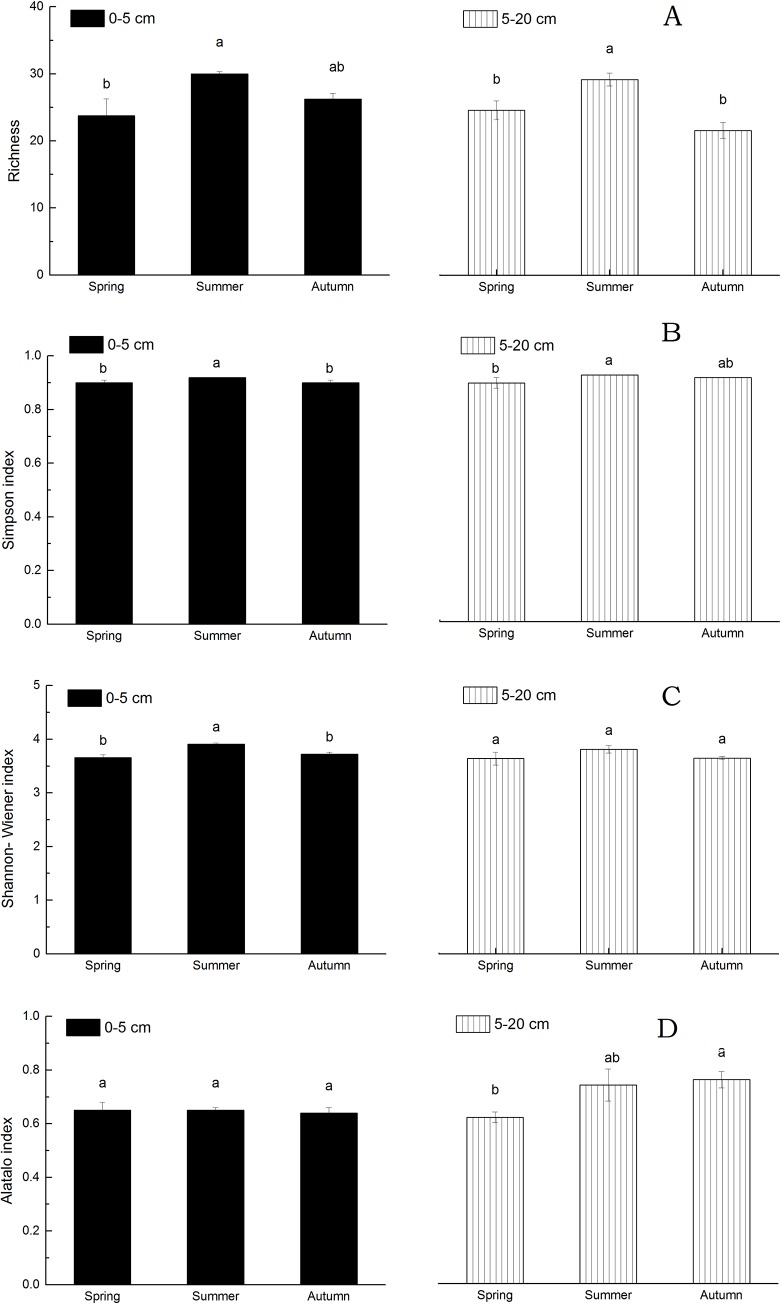
Microbial diversity in terms of richness (A), the Simpson index (B), the Shannon-Wiener index (C), and the Alatalo index (D) in orchard soil at depths of 0–5 and 5–20 cm in different seasons. Bars with the same letter(s) are not significantly different between seasons at each depth at *P* < 0.05 based on LSD post hoc tests.

The microbial diversity analysis of the orchard soil showed that the richness (number of microbial species) was in the range of 18 to 31, and the Simpson index, Shannon-Wiener index and Alatalo index ranged from 0.76 to 0.93, 3.16 to 3.97 and 0.59 to 0.76, respectively (Fig 2). The microbial diversities of the orchard soil in different seasons varied. At the 0-5-cm soil depth, the richness and the Simpson, Shannon-Wiener and Alatalo indexes peaked in the summer, with values of 30.0, 0.92, 3.91 and 0.65, respectively. The Simpson and Shannon-Wiener indexes in the summer were significantly different from those in the spring and autumn (*P <* 0.05). A significant difference in richness found between the summer and spring. No significant differences in the Alatalo index observed between seasons (*P >* 0.05). In the 5-20-cm soil layer, the largest values of richness and the Simpson and Shannon-Wiener indexes were still found in the summer, with values of 29.2, 0.92 and 3.81, respectively. The richness and the Simpson index of the soil microbes in the summer were significantly greater than those in the spring (*P <* 0.05). There were no significant differences in the Shannon-Wiener index. However, the Alatalo index of the soil microbes in autumn (0.76) was significantly greater than that in the spring (0.62) (*P <* 0.05). Overall, the microbial diversity of the orchard soil in the summer was greater than that in the spring and autumn.

### 3.4. Correlation between microbial communities and soil properties

Correlation analysis was carried out between the soil properties (pH, TOC, TN, C/N ratio, CEC, and exchangeable K^+^, Na^+^, Mg^2+^ and Ca^2+^), microbial quantities (total PLFAs, bacterial PLFAs, fungal PLFAs, actinomycetic PLFAs, and B/F ratio) and microbial diversities (richness and the Simpson, Shannon-Wiener and Alatalo indexes). The results showed that the microbial quantities (total PLFAs, bacterial PLFAs, fungal PLFAs, and actinomycetic PLFAs) were significantly negatively correlated with the pH, TOC, TN and CEC, with Spearman correlation coefficients of -0.530 to -0.618 (*P* < 0.01), -0.572 to -0.642 (*P* < 0.01), -0.401 to -0.422 (*P* < 0.05) and -0.791 to -0.831 (*P* < 0.01), respectively (Table 5). The microbial diversity (richness and the Simpson and Shannon-Wiener indexes) was significantly negatively correlated with the pH, TOC, TN and CEC, with Spearman correlation coefficients of -0.460 to -0.753 (*P* < 0.05), -0.419 to -0.707 (*P* < 0.05), -0.450 to -0.526 (*P* < 0.05), and -0.446 to -0.722 (*P* < 0.05), respectively (Table 6).

**Table 6:** Spearman correlation coefficient matrix for microbial community variation and chemical properties in orchard soils.

Item	pH	TOC	TN	C/N ratio	CEC	Exchangeable			
						K^+^	Na^+^	Mg^2+^	Ca^2+^
Total PLFAs	-0.614**	-0.617**	-0.401*	-0.302	-0.817**	0.284	0.493**	0.119	-0.177
Bacterial PLFAs	-0.618**	-0.642**	-0.422*	-0.317	-0.831**	0.296	0.485**	0.141	-0.160
Fungal PLFAs	-0.530**	-0.572**	-0.401*	-0.313	-0.791**	0.390*	0.482**	0.229	-0.074
Actinomycetal PLFAs	-0.593**	-0.555**	-0.434*	-0.248	-0.744**	0.320	0.525**	0.150	-0.165
B/F ratio	-0.517**	-0.565**	-0.354	-0.119	-0.595**	-0.259	0.131	-0.189	-0.263
Richness	-0.460*	-0.419*	-0.526**	0.051	-0.446*	0.403*	0.622**	0.332	-0.085
Simpson diversity	-0.753**	-0.707**	-0.498**	-0.159	-0.722**	0.132	0.517**	-0.012	-0.328
Shannon-Wiener diversity	-0.602**	-0.446*	-0.450*	0.007	-0.556**	0.330	0.504**	0.090	-0.380*
Alatalo evenness	-0.054	0.096	0.215	-0.062	0.028	-0.176	-0.252	-0.203	-0.092

PLFA = phospholipid fatty acid, B/F ratio = ratio of bacterial to fungal PLFAs, TOC = total organic carbon, TN = total nitrogen, CEC = cation exchange capacity, C/N ratio = ratio of total organic carbon to total nitrogen.

* and ** indicate Spearman coefficient significant differences at *P <* 0.05 and 0.01, respectively (*n* = 28).

## Discussion

### 4.1. Variation in the microbial population in the soil ecosystem among seasons

**Fig 3:**
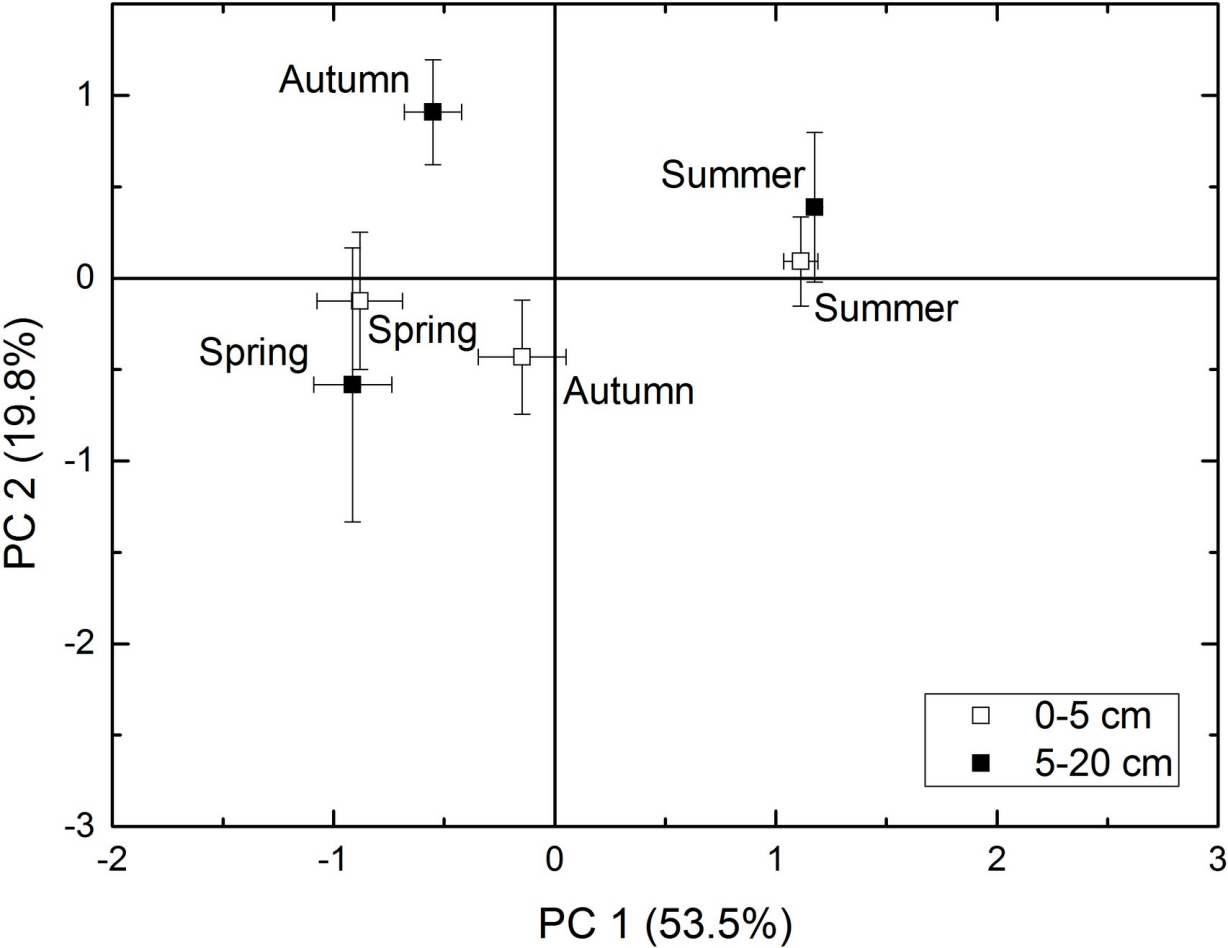
Principal component analysis (PCA) of the microbial community in orchard soil sampled in different seasons and at different depths. The percent variance explained by each component (PC) is shown in parentheses. Error bars represent standard errors (*n* = 28).

Usually, the microbial population varies based on moisture and temperature changes among seasons. However, whether this change could be easily detected depends on how long the suitable season lasts and the degree to which extreme water and temperature conditions affect the soil microorganisms. In the subtropical mountain area examined in this study, the soil was used to plant peach, and obvious microbial changes were confirmed. Principal component 1 explained 46.8% of the variation in the soil microbial community (Fig 3). The total, bacterial, fungal, and actinomycetic PLFAs, the B/F ratio, and richness were the main factors associated with principal component 1 (S1 Table). The remarkable change in the microbial community related to the peak soil microorganism growth in the summer due to permissive temperatures and rainfall.

Principal component 2 explained 26.5% of the variation in the soil microbial community (Fig 3). The Simpson index and Shannon-Wiener index were the major associated factors (S1 Table). This result could be explained by the microorganisms propagating well and maintaining a good balance in the summer in the tested orchard soil system.

The results of our study are consistent with the results of Zhu et al. [14] from an evergreen broadleaf forest and Qi et al. [15] from a bamboo grove in the subtropical climate zone. Investigations of an orchard [13], grassland [12] and forest [17] in the temperate monsoon climate zone reported similar trends. However, unlike our results, Shi et al. [16] observed seasonal variation characterized by low values for most of the microbial biomass values (C, N, and P), enzyme activities and PLFAs in the summer in southwestern Quebec, Canada. This difference related to the particularly dry climate (drier than the long-term average of the season) with a significantly lower moisture content than in the spring and autumn in our system. It is well documented that temperature and moisture are the main factors related to microbial abundance and distribution.

### 4.2. Relationship between soil physicochemical properties and microbial communities

The reported relationship between soil physicochemical properties and microbial communities varies among studies. In this study, in terms of the distribution among seasons and between soil depths, the total PLFAs, bacterial PLFAs, fungal PLFAs, actinomycetic PLFAs, number of species (richness), and Simpson and Shannon-Wiener diversity indexes were significantly negatively correlated with the soil TOC, TN and CEC. In the summer, most soil microbes experienced rapid growth, and a significant decrease in the soil pH, TOC, TN, and CEC was found (Table 1). Liu et al. [18] also found the microbial dominance index and Shannon-Wiener index to be negatively related to soil NH_4_^+^-N and NO_3_^—^N in an apple system based on observations during different growing periods. However, in terms of change evaluated based on utilization, Yao et al. [6] illustrated that microbial biomass C, basal respiration and total PLFAs were strongly correlated with organic C and TN in a red soil-orchard ecosystem. In some ecosystems, soil microbes grow rapidly via energy and nutrition consumption [25, 26]. This study clearly suggested that the propagation and growth of soil microorganisms from spring to summer require energy from TOC and nutrition from N in a subtropical orchard system. Microbes mainly act as “consumers”. In most microbial groups, PLFAs did not vary significantly between soil horizons but did vary between seasons. Microbial richness and the Simpson and Shannon-Wiener indexes were all highest in summer. Microbial diversity was negatively correlated with major soil chemical properties.

In fact, the triggers of microbes rapid propagation most likely are high moisture, warm temperature and outside carbon input (S2 Table). But the factors of decreases of 0–20 cm soil TOC, TN and CEC are quite complicated. Some of microbes happily consume outside carbon and nitrogen, someone likely eat nutrition matters in soil, and some of them get energy from metabolites of other microbial groups. Most importantly, several key microbial organisms living habits even effect the whole interaction between microbial community dynamics and soil chemical properties. It is hardly conclusive because that the function of many key soil microbes remain unclear, So further study is needed on the mechanism of the negative relationship between microbial size and diversity of all components of the microbial community and TOC, TN and cation exchange capacity.

## Conclusions

In subtropical orchards, the temperature and humidity in the summer are conducive to the growth of soil microorganisms. PLFA analysis showed that the quantity of soil microbes in the soil samples collected in the summer was significantly higher than that collected in the spring and autumn. The Simpson and Shannon-Wiener indexes also peaked in the summer samples. The total PLFAs, bacterial PLFAs, fungal PLFAs, actinomycetic PLFAs, richness, and Simpson and Shannon-Wiener indexes were significantly negatively correlated with seasonal changes in the soil pH, TOC, TN and CEC.

The function of the microbial community in the translation and accumulation of soil nutrients from summer to autumn should be merited further study. The changes in functional flora (including Archaea) in the soil resulting from different orchard management strategies merit further investigation, and the relationships between the change in flora and the orchard litter, soil organic matter (SOM), soil C/N ratio, ammonia N, nitrate N, pH, soil respiration, and other parameters should be further studied.

## Supporting information

S1 Table Principle component (PC) factor values and total eigenvector coefficients between PC factors and microbial community indexes after varimax rotation. (DOC)

S2 Table Carbon input of peach litters in different seasons. (DOCX)
