# Supplementary material for: Seasonal Change in Microbial Diversity and Its Relationship with Soil Chemical Properties in an Orchard
Source: PLoS One. 2019 Dec 31;14(12):e0215556. doi: 10.1371/journal.pone.0215556 (PMC6938340; doi:10.1371/journal.pone.0215556)
Supplement: S1 Table — (DOC) [file pone.0215556.s001.doc]

**Supplemental Information**

**Table S1**

Principle component (PC) factor values and total eigenvector coefficients between PC factors and microbial community indexes after varimax rotation

| PC | Value | Contribution rate | |  | Factor | | | | | | | | |
| --- | --- | --- | --- | --- | --- | --- | --- | --- | --- | --- | --- | --- | --- |
|  |  | Pr Var | Cum Var |  | Total | Bacterial | Fungal | Actinomy-cetal | B/F ratio | Richness | Simpson | Shannon-Wiener | Alatalo |
|  |  | …%… | |  | ………PLFAs……… | | | |  | …………Indexes………… | | | |
| 1 | 5.35 | 53.50 | 53.50 |  | 0.96 | 0.97 | 0.95 | 0.96 | 0.30 | 0.81 | 0.62 | 0.56 | 0.35 |
| 2 | 1.98 | 19.80 | 73.30 |  | -0.09 | -0.07 | -0.14 | -0.14 | 0.40 | -0.36 | 0.71 | 0.54 | 0.89 |

PC= principle component, Pr Var= principle variance, Cum Var= cumulative variance, PLFA=phospholipid fatty acid, B/F ratio =ratio of bacterial to fungal PLFAs.

Factor loadings |*x*| > 5% of total eigenvector coefficients after varimax rotation.
