# Supplementary material for: Seasonal Change in Microbial Diversity and Its Relationship with Soil Chemical Properties in an Orchard
Source: PLoS One. 2019 Dec 31;14(12):e0215556. doi: 10.1371/journal.pone.0215556 (PMC6938340; doi:10.1371/journal.pone.0215556)
Supplement: S2 Table — (DOCX) [file pone.0215556.s002.docx]

**Table S2**

Carbon input of peach litters in different seasons (g m^-2^)

| Tree Cases | Spring | | | Summer | | | Autumn | | |
| --- | --- | --- | --- | --- | --- | --- | --- | --- | --- |
|  | Apr | Mar | Jun | Jul | Aug | Sep | Oct | Nov | Dec |
| No. 1 | 0.29 | 3.63 | 0.46 | 1.45 | 6.17 | 7.92 | 6.68 | 1.71 | 0.00 |
| No. 2 | 0.54 | 5.13 | 1.98 | 3.32 | 12.02 | 6.07 | 9.49 | 0.33 | 0.00 |
| No. 3 | 0.48 | 4.58 | 2.04 | 3.96 | 7.42 | 13.30 | 4.89 | 0.82 | 0.00 |
| No. 4 | 0.27 | 4.24 | 1.30 | 2.62 | 8.25 | 3.38 | 5.71 | 0.60 | 0.00 |
| No. 5 | 0.56 | 5.10 | 1.49 | 3.06 | 10.18 | 3.27 | 6.12 | 0.64 | 0.00 |
| No. 6 | 0.50 | 3.22 | 1.80 | 4.52 | 12.39 | 3.23 | 4.78 | 0.69 | 0.00 |
| Means | 0.44 | 4.32 | 1.51 | 3.15 | 9.41 | 6.19 | 6.28 | 0.80 | 0.00 |

Peach litter were collected every month by set nylon net of 4 m×4 m with 20 mesh under trees, 6 replicates. Dry matter input data is average of 6 replicates, carbon content of dry litter samples was tested after the drying condition of 12 hours at 85℃.
